# Vitamin D in the Transition from Acute to Chronic Pain: A Systematic Review

**DOI:** 10.3390/nu17111912

**Published:** 2025-06-01

**Authors:** Diana Marisol Abrego-Guandique, Sara Ilari, Saverio Nucera, Lucia Carmela Passacatini, Erika Cione, Roberto Cannataro, Luca Gallelli, Maria Cristina Caroleo, Vincenzo Mollace, Carolina Muscoli

**Affiliations:** 1 Department of Health Sciences, University “Magna Graecia” of Catanzaro, 88100 Catanzaro, Italy; dianamarisol.abregoguandique@unicz.it; 2 Department for the Promotion of Human Sciences and Quality of Life, San Raffaele Roma University, 00166 Rome, Italy; sara.ilari@sanraffaele.it; 3 Laboratory of Physiology and Pharmacology of Pain, IRCCS San Raffaele Roma, 00166 Rome, Italy; carmela.passacatini@sanraffaele.it; 4 Department of Health Sciences, Institute of Research for Food Safety and Health (IRC-FSH), University “Magna Graecia” of Catanzaro, 88100 Catanzaro, Italy; mollace@unicz.it (V.M.); muscoli@unicz.it (C.M.); 5 Department of Pharmacy, Health and Nutritional Sciences, University of Calabria, 87036 Rende, Italy; erika.cione@unical.it; 6 Galascreen Laboratories, University of Calabria, 87036 Rende, Italy; rcannataro@nutrics.it; 7 Research Division, Dynamical Business & Science Society, DBSS International SAS, Bogota 110311, Colombia; 8 Operative Unit of Pharmacology and Pharmacovigilance, “Renato Dulbecco” University Hospital, 88100 Catanzaro, Italy; luca.gallelli@unicz.it; 9 Research Center FAS@UMG, Department of Health Science, University “Magna Graecia” of Catanzaro, 88100 Catanzaro, Italy

**Keywords:** vitamin D, acute pain, acute to chronic pain transition, chronic pain prevention

## Abstract

Background: The transition from acute to chronic pain is an important clinical phenomenon that significantly impacts the healthcare system. Despite decades of research, preventing this transition remains a complex challenge. Many studies have explored the various factors that contribute to the development of chronic pain, but the underlying mechanisms are still largely unclear. In this frame, vitamin D (VD) plays an important role in pain mechanism development, with emerging evidence suggesting it influences pain perception, inflammation, and nerve function. Methods: A total of 14 eligible original research articles were identified. Results: Our qualitative analysis showed that VD did not directly influence the transition from acute to chronic pain, but it affected pain intensity, improving outcomes in patients at risk of developing chronic pain. Conclusions: Additional randomized clinical trials, particularly double-blind, placebo-controlled studies, which are regarded as the gold standard in clinical research, are warranted to evaluate the role of vitamin D in the progression from acute to chronic pain

## 1. Introduction

Chronic pain is still a challenging task in clinical practice. About 30% of adults worldwide suffer from it [1], resulting in a poor quality of life, a higher risk of developing disabilities, and a social impact [2,3]. The therapeutic approach is still aimed at determining which drugs or drug combinations offer the most efficacy with the fewest adverse effects in order to improve patient compliance, even though available treatments often produce insufficient pain relief [4]. The difficulty in the pharmacological management of chronic pain derives from the complexity of the pain neuroaxis [5]. In addition, several cellular and molecular events, such as considerable transcriptional activity, also contribute to persistent pain [6]; thus, pain transmission must be seen as a multifactorial dynamic process. In the absence of disease-modifying therapies and proper symptomatic treatment, preventive measures are crucial. Over the past few years, the process known as the transition from acute to chronic pain has gained increasing attention. It has been proposed that some chronic pain forms could be considered as a progression of acute pain, leading to a shift from physiological to pathological pain [6]. This conceptualization, although not widely accepted [7], suggests that one mechanistic type of pain can evolve into another kind of pain. For instance, acute pain caused by tissue injury and inflammation can develop into neuropathic pain. The development of chronic pain frequently occurs as a side effect of chemotherapy and radiotherapy [8,9] or is associated with surgical tissue trauma. In this case, nerve damage constitutes a significant determinant of this process, but the mechanisms, including those at the cellular and molecular levels, remain to be elucidated. The research focuses on the synaptic [10] and immune [11,12] adaptive responses after end-organ damage as well as on deficits in neuronal energy balance [13,14]. Within this framework, a recent study has demonstrated that N-Acylethanolamine acid amidase (NAAA) acts as a regulatory checkpoint for spinal metabolism, resulting in a significant impact on the pain consolidation process. Notably, this enzyme can be targeted by small-molecule therapeutics with inhibitory activity, thereby paving the way for a novel therapeutic approach in the progression of chronic pain [15]. In the clinical setting, the timely identification of individuals who are prone to developing chronic pain is essential to minimize the risk of the transition into chronic intractable pain. While injury, acute stress events, surgery, metabolic diseases, chemotherapy, and infections are well-established trigger factors [16], the precise timing of transition remains an open question. However, predictive factors such as patient demographics, phenotypic trait variations, acute pain characteristics (i.e., acute pain intensity/severity, duration, and cumulative trauma exposure) [17,18,19], and psychosocial factors [20] have been identified. It is commonly accepted that this process occurs some period after the onset of acute pain. Still, the possibility that both acute and chronic pain mechanisms may arise simultaneously in individuals with persistent pain cannot be ruled out [16]. Another important concern relates to the management of acute pain conditions. Indeed, treatment is largely dependent on a handful of analgesic drug classes, such as opioids, which may lose effectiveness over time and can also lead to addiction. Recently, the literature has placed a significant focus on the interplay between vitamin D (VD) and pain. VD, known as a hormone and neuroactive steroid, can exert analgesic effects by modulating neuronal excitability. The molecular mechanisms by which it interferes with nociceptive processing encompass the inhibition of both nitric oxide synthase and Cox-2 expression, stimulation of 15-prostaglandin dehydrogenase (15-PGDH) expression, and upregulation of transforming growth factor beta in glial cells and macrophage colony-stimulating factor in astrocytes and microglia [21]. Observational studies indicate that hypovitaminosis D is associated with musculoskeletal pain [22], myalgia [23], chronic lower back pain, and chronic headache [24]. Additionally, appropriate VD supplementation, particularly in patients with VD deficiency, can enhance pain relief in several chronic pain conditions [25]. In this systematic review, we examine the impact of VD status and its supplementation in the transition from the acute to chronic pain state in humans. To the best of our knowledge, no existing systematic review or meta-analysis has yet offered a qualitative assessment of the relationship between vitamin D and the process of pain chronification.

## 2. Methods

This systematic review followed the Preferred Reporting Items for Systematic Reviews and Meta-Analyses (PRISMA) guidelines [26] and was registered with the PROSPERO International Prospective Register of Systematic Reviews (CRD42024563201). A semi-automated methodology was employed using the MySLR platform, available at https://myslr.unical.it (accessed on 1 July 2024). This digital tool simulates human reasoning by utilizing the Latent Dirichlet Allocation (LDA) algorithm for topic modeling [27], a method previously applied in other studies [26,27,28,29].

### 2.1. Paper Location and Selection

Two independent reviewers (S.I. and D.M.A.-G.) conducted comprehensive literature searches using PubMed, Scopus, and Web of Science to locate peer-reviewed articles published prior to 1 June 2024. This systematic review encompasses clinical research focused on vitamin D (VD) levels in relation to the transition from acute to chronic pain. The search strategy included keywords such as “VD and chronicization pain”, “VD and postoperative pain”, “VD and neuropathic pain”, “chronicization pain and integra-tion of VD”, “VD and chronic pain development”, “VD and “post-surgical pain” OR “postoperative analgesia” OR “post-surgical analgesia” OR “post-operative pain”, “VD and chemotherapy pain”, “VD and pain transition”.

### 2.2. Study Selection and Data Extraction

Studies were included in the systematic review focusing on the role of VD in the transition of acute pain in chronic pain, which were all published before 1 June 2024.

Types of Study: randomized clinical trials or prospective (cross-sectional), case–control, or longitudinal studies.

The eligibility criteria for including studies in this systematic review were defined as follows: studies that highlighted the transition from acute to chronic pain in relation to the treatment administered, its duration, and the outcomes achieved; studies that were conducted in humans aged 18 years or older that reported on acute or subacute pain and either assessed vitamin D supplementation or reported serum vitamin D levels, provided they included sufficient information on pain assessment scales. Only articles in English and published between 2010 and 2024 were considered.

The following exclusion criteria were applied during the selection process: publications not written in English; reviews, meta-analyses, systematic reviews, letters, conference abstracts, commentaries, book chapters, or proceedings; studies involving animal models or in vitro experiments; research not focused on acute or subacute pain; studies that did not evaluate vitamin D levels; and those lacking essential data, including information on pain assessment scales.

Two authors, S.I. and D.M.A.G., independently screened all titles and abstracts for eligibility. Any disagreements that arose during the selection process were resolved through discussion, with the goal of reaching a consensus. When necessary, a third reviewer (S.N.) was involved. One author (D.M.A.-G.) extracted the data from the included studies and two others (S.I. and S.N.) subsequently verified it.

The extracted data included author names, year of publication, country of study, study design, participant details (sample size, age, and gender), intervention or vitamin D levels, pain-related outcomes, and the main findings.

### 2.3. Quality Assessment and Risk of Bias Assessment

The risk of bias in the design and analysis of each included study was independently evaluated by two reviewers (D.M.A.-G. and S.I.) using the NIH Study Quality Assessment Tool (accessed 10 June 2023) [27]. When necessary, three additional reviewers (S.N., C.M., and M.C.C.) were consulted to reach a consensus. The evaluation utilized the Quality Assessment Tool for Observational Cohort and Cross-Sectional Studies, the Quality Assessment Tool for Case–Control Studies, and the Quality Assessment Tool for Controlled Intervention Studies. These instruments, each comprising 12 to 14 questions, are designed to guide the assessment of internal validity by examining factors such as selection bias, methodological rigor, information accuracy, measurement consistency, and potential confounders.

## 3. Results

### 3.1. Data Collection

After multiple screening phases, 14 articles addressing vitamin D (VD) levels or VD supplementation during the transition from acute to chronic pain were included in the qualitative analysis (systematic review).

The systematic review identified 2816 records from the literature search. After removing duplicates, 1583 articles were left for further examination. Of these, 1616 were excluded based on their title and 471 articles were excluded by abstract. After screening by title and abstract, 529 articles were considered for full-text eligibility assessment.

A total of 360 articles were excluded from the final database because they were reviews, 37 were excluded because they were in vitro and in vivo studies, and 118 articles were excluded because they were systematic reviews.

Finally, 14 clinical trials involving patients, published between 2010 and 2023, met the eligibility criteria and were included in our review.

**Figure 1:**
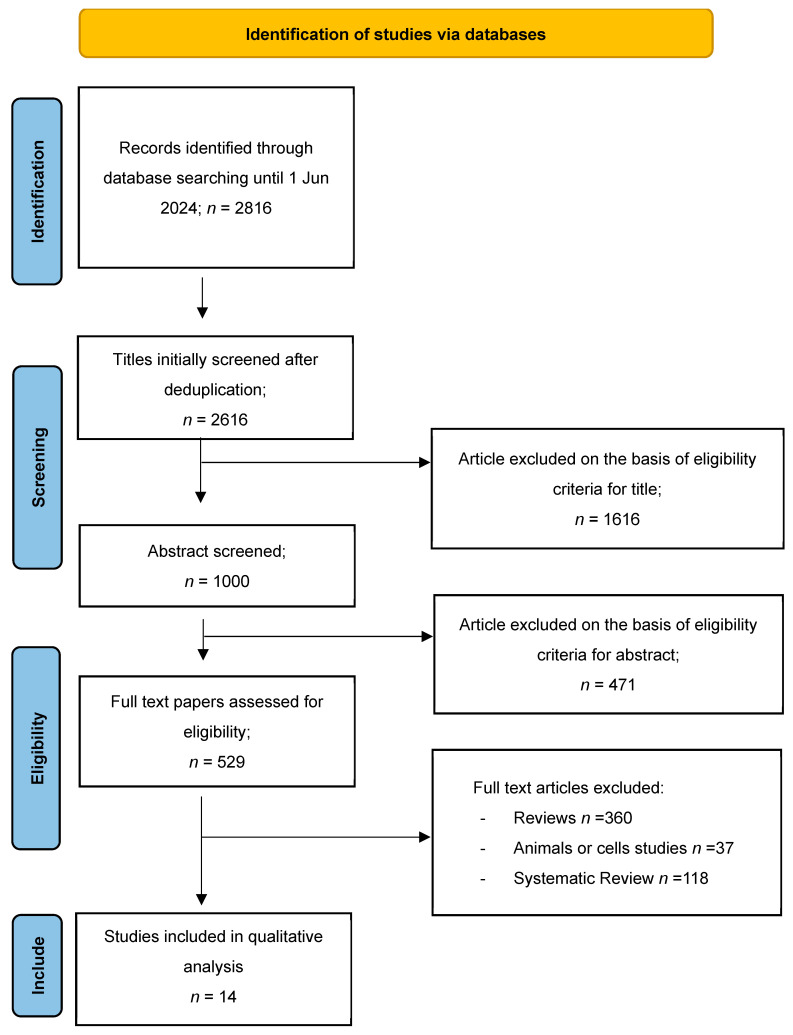
PRISMA flow diagram illustrating the selection algorithm for eligible studies.

The literature search and screening process is detailed in Figure 1.

### 3.2. Characteristics of the Studies Included

Table 1 summarizes the characteristics of studies reporting blood levels of VD and its role in the transition from acute to chronic pain, while Table 2 summarizes those of the studies also reporting VD supplementation. Articles in both tables appear in descending order by year of publication.

Of the fourteen eligible studies, nine observational studies were included: five prospective or retrospective studies, one case–control study, one cross-sectional study, and two longitudinal studies. In addition, five randomized intervention studies were also included. The studies were published from 2010 to 2023 and conducted in seven countries: USA, Europe, China, India, Iran Czech Republic, Poland, Spain, and Hong Kong. The ages of all the participants ranged from 30 to 100 years. Seven studies were conducted in both sexes [30,31,32,33,34,35,36], whereas one study was exclusively conducted in males [37] and five in females [38,39,40,41]. One study did not report the sex [42]. VD levels or supplementation were assessed in several conditions such as pre/post-surgery [30,33,34,35]; patients under chemotherapy, including paclitaxel [32,38,39]; women with an aromatase inhibitor [36,40,41,43]; non-chronic musculoskeletal pain [31,42]; and middle age and elderly subjects [37].

This systematic review included studies that utilized a wide variety of pain test batteries, such as the Numeric Rating Scale (NRS) [30], the Visual Analog Scale (VAS) [31,33,34,35,42,43] for the self-reported level of pain intensity, a 5-point Likert scale [33] for the self-reported level of pain relief, and the Brief Pain Inventory (BPI) Short Form for measuring both pain intensity and pain interference in the patient’s life [36]. The location and duration of pain were assessed using the Painful Sites questionnaire [35] and the McGill Pain Map [29], whereas functional disability was recorded in accordance with the Modified Oswestry Disability Questionnaire (MODQ) [31]. MNSI (Michigan Neuropathy Screening Instrument) [32], WOMAC (Western Ontario and McMaster Universities Osteoarthritis Index) [33], and sensory CIPN (chemotherapy induced peripheral neuropathy) were used as disease-specific scales [38,39]. Finally, FIQ (Fibromyalgia Impact Questionnaire) [36], HAQ (Health Assessment Questionnaire—Disability) [36,40,41], and CIPN20 (Chemotherapy-Induced Peripheral Neuropathy Quality of Life questionnaire) were used in several studies to assess the impact of pain in relation to physical function, the extent of the patient’s functional ability, and quality of life, respectively, as reported in Table 1 and Table 2.

**Table 1:** Characteristics of the studies included.

Study/ Year	Type of Study	Country	Population	Mean, Age, Years (SD/IC)	Sex	*n*	Intervention/ Duration	Levels of VD	Pain Test Used
Chen et al., 2023 [38]	Prospective-retrospective study	USA	Patients with early-stage breast cancer with paclitaxel	51.1 y ± 9.9	F	1191	NR	1.57 (1.14–2.15) IC	Sensory CIPN
Zeng et al., 2022 [30]	Retrospective cohort study	China	Patients who underwent elective non-cardiac thoracic surgery	Group 1 57.5 y ± 13.1 Group 2 58.8 y ± 11.8	F = 74 M = 61	135 Group 1 = 73 Group 2 = 62	3 months	Group 1 low 25(OH)D levels (<30 nmol/L) Group 2 25(OH)D levels (>30 nmol/L)	Pain scores (numerical rating scale)
Hao-Wei et al., 2021 [42]	Cross-sectional study	China	Non-specific acute lower back pain (Ns-ALCP) and non-specific-chronic lower back pain patients (Ns-CLBP)	63.42 ± 11.26 years, with a range of 33 to 80 years.	NR	198 Ns-ALCP = 60 Ns-CLBP = 78 Control = 60	NR	Ns-ALBP 21.44 ± 8.46 Ns-CLBP 18.25 ± 8.05 (ng/mL) Control 25.70 ± 10.04	VAS scale
Jennaro et al., 2020 [39]	Prospective observation clinical study	USA	Patients with stage I–III breast cancer receiving weekly paclitaxel	47.6 y (28–59 IC) 54.6 y (34–71 IC)	F	37	Duration 12 weeks	VD deficiency (defined as <20 ng/mL) was identified in 41% (15/37) of assessed patients	CIPN20
Panwar et al., 2018 [31]	Prospective, observational, triple arm, case and control study	India	Patients with lower back pain of duration ≥ 6 weeks (CLBP), patients with subacute lower back pain (SLBP) and controls.	CLBP 40.40 y ± 13.638 SLBP 40.69 y ± 14.469 Control 37.95 y ± 15.255	CLBP M = 104 F = 146 SLBP M = 82 F = 95 Control M = 112 F = 136	675 CLBP = 250 SLBP = 177 Control = 248	3 months	CLBP 20.36 ± 12.569 SLBP 21.42 ± 13.209 Controls 20.84 ± 6.931	BMcGill Pain Map VAS scale MODQ
Grim et al., 2017 [32]	Case and control	Czech Republic	Patients with breast carcinoma from the undergoing chemotherapy based on 80 mg/m^2^ paclitaxel on a weekly basis (12 cycles)	56 y ± 12.2	M = 10% F = 90%	70	Duration 12 weeks Evaluation 1 after 4 weeks Evaluation 2 End treatment 12 weeks	Before chemotherapy Without NP 38.08 ± 15.6 nmol/L With NPz 26.94 nmol/L ± 8.5 During chemotherapy (4 week) Without NP 37.44 nmol/L ± 19.9 With NP 24.28 nmol/L ± 8.9 After chemotherapy (12 week) Without NP 42.33 nmol/L ± 9.6 With NP 31.4 nmol/L ± 10.4	MNSI
McCabe et al., 2016 [37]	Multicenter/longitudinal study	Europe	Population sample of middle age and elderly men	aged 40–79	M	2736	Participants were invited to attend repeat assessment after a mean interval of 4.3 years (range 3–5.7 years).	25(OH)D–quintiles (ng/mL) 1. ≥36.3 2. 26.7–36.2 3. 20.7–26.6 4. 15.6–20.6 5. <15.6 25(OH)2D–quintiles (pg/mL) 1. ≥72.5 2. 62.2–72.5 3. 55.2–62.0 4. 45.4–55.0 5. <45.4	Painful sites
Lee et al., 2015 [33]	Longitudinal cohort study	Hong Kong	Patients after surgery knee arthroplasty	62–73 y	F = 153 M = 61	214	3 months	25(OH)D levels <30 nmol/L >30 nmol/L–<50 nmol/L >50 nmol/L	Pain scale WOMAC EQ-5D-VAS

Abbreviations: CIPN, chemotherapy-induced peripheral neuropathy; CIPN20, 20-item Quality of Life Questionnaire for Chemotherapy-Induced Peripheral Neuropathy; CLBP, chronic lower back pain; EQ-5D-VAS, EuroQol 5-Dimension Visual Analog Scale; IC, interquartile range; MNSI, Michigan Neuropathy Screening Instrument; MODQ, Modified Oswestry Disability Questionnaire; NR, not reported; Ns-ALBP, non-specific acute lower back pain; Ns-CLBP, non-specific chronic lower back pain; SLBP, subacute lower back pain; VAS, Visual Analog Scale; VD, vitamin D; WOMAC, Western Ontario and McMaster Universities Osteoarthritis Index.

**Table 2:** Characteristics of the studies included.

Study/Year	Type of Study	Country	Population	Mean, Age, Years (SD/IC)	Sex	*n*	Intervention/ Duration	Levels of VD	Pain Test Used
Melika et al., 2019 [34]	Randomized clinical trial	Iran	Adult patients with diagnosed brain tumor with serum level of 25 (OH) VD ≤ 20 ng/dL	VD 48.2 ± 15.3 y PL 44.3 ± 15.2 y	M = 30 F = 30	60 VD = 30 PL = 30	300,000 IU VD 2- to 14-day interval before surgery	Preoperatory VD = 15.9 ± 3.8 PL = 14.5 ± 3.6	VAS scale
Krasowska et al., 2019 [35]	Double-blind randomization study	Poland	Patients undergoing posterior lumbar interbody fusion (PLIF) followed by rehabilitation.	VD 41.92 ± 2.97 PL 47.33 ± 2.15	VD M = 9 F = 9 PL M = 9 F = 12	39 VD = 18 PL = 21	VD (3200 IU dose of VD/day for 5 weeks) and placebo group (PL)	The initial serum 25(OH)D3 (nmol/L) VD 46.63 ± 1.69 PL 55.71 ± 4.11 after 5 weeks VD 75.03 ± 3.03 PL 53.62 ± 3.07 After surgery VD normal PL decrease levels	VAS scale
Niravath et al. (2019) [40]	Randomized control trial	USA	Post-menopausal women who were beginning adjuvant aromatase inhibitor therapy	64 y (44–82 y)	F = 93	93 High dose = 46 Standard dose = 47	Standard-dose VD3 (800 IU daily for 52 weeks), or high-dose VD3 (50,000 IU weekly for 12 weeks, followed by 2000 IU daily for 40 weeks)	Baseline vitamin D levels averaged 24.2 ng/mL in the standard-dose group and 21.7 ng/mL in the high-dose group. After 12 weeks, levels rose to 29.3 ng/mL and 50 ng/mL, respectively.	HAQ-II
Prieto-Alhambra et al. (2011) [43]	Prospective cohort study	Spain	Women with breast cancer who were women starting aromatase inhibitor therapy	VD < 30 ng/mL 2.63 (8.77) VD > 30 ng/mL 60.20 (9.64)	F	284 VD < 30 ng/mL = 251 VD > 30 ng/mL = 33	All received daily VD (800 IU) with calcium. Women with baseline VD concentration < 30 ng/mL also received 16,000 IU of D3 orally every 2 weeks. 3 months	Following baseline assessment, 89.7% (260 participants) were vitamin D deficient (<30 ng/mL), with 18.5% (48 individuals) showing severe deficiency (<10 ng/mL)	VAS scale
Rastelli et al. (2011) [36]	Randomized control trial	USA	Patients with musculoskeletal pain in women receiving adjuvant anastrozole improves aromatase inhibitor	61.5 (8.4)	F = 57%	57 VD = 28 PL = 29	50,000 IU VD2 weekly for 8 weeks then monthly for 4 months; or 50,000 IU VD2 weekly for 16 weeks then monthly for 2 months.	Stratum A = 20–29 ng/mL Stratum B = 10–19 ng/mL	BPI-SF FIQ HAQ-DI
Khan et al. (2010) [41]	Randomized, placebo-controlled trial	USA	Women with early-stage, receptor-positive, invasive breast cancer who were candidates for adjuvant aromatase inhibitor therapy	Mean age 56 y	F = 60	60	Group 1: VD (high dose) Group 2 (control): VD (standard dose) 50,000 IU of VD weekly (16 weeks)	<40 ng/mL vit D = 47 >40 ng/mL vit D = 13	HAQII

Abbreviations: 25(OH)D, 25-hydroxyvitamin D; BPI-SF, Brief Pain Inventory–Short Form; FIQ, Fibromyalgia Impact Questionnaire; HAQ-DI, Health Assessment Questionnaire–Disability Index; HAQ-II, Health Assessment Questionnaire II; IC, interquartile range; PL, placebo; PLIF, posterior lumbar interbody fusion; VAS, Visual Analog Scale; VD, vitamin D; VD2, vitamin D2.

### 3.3. Quality Assessment and Risk of Bias Assessment

**Figure 2:**
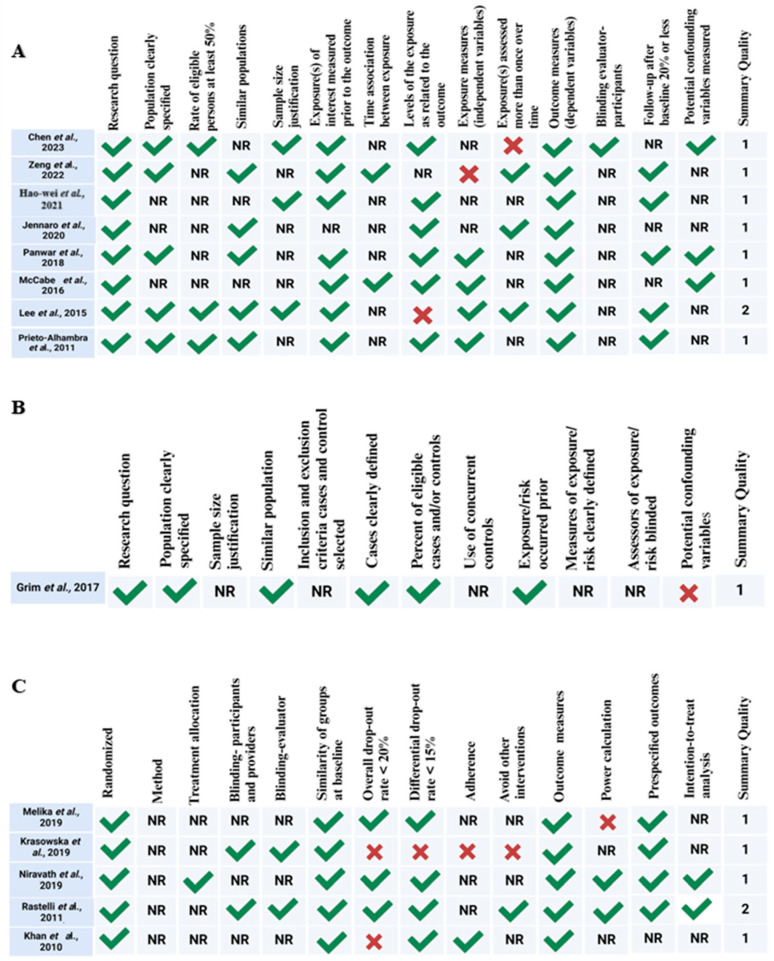
(**A**) Summary of risk-of-bias assessment according to the National Institutes of Health Quality Assessment Tool for Observational Cohort and Cross-Sectional Studies (NIH, 2014) [30,31,33,37,38,39,42,43]. (**B**) Summary Quality Assessment of Case–Control Studies [32]. (**C**) Summary Quality Assessment according to Health Quality Controlled Intervention Studies (NIH, 2014) [34,35,36,40,41]. The quality rating is 0 for poor (0–4 out of 14 questions), 1 for fair (5–9 out of 14 questions), or 2 for good (>10 out of 14 questions). NR: not reported; created with BioRender (https://www.biorender.com/ accessed on 1 July 2024).

The risk of bias assessment is summarized in Figure 2A–C. As mentioned above, we used three tools: the Quality Assessment Tool for Observational Cohort and Cross-Sectional Studies (n = 8), the Quality Assessment of Case–Control Studies (n = 1), and the Quality Assessment of Controlled Intervention Studies (n = 5). Using these tools, out of the fourteen studies included in the systematic review, two studies were rated as “good” [33,36] and twelve as “fair” [30,31,32,34,35,37,38,39,40,41,42,43,44]. Among the eight observational cohort and cross-sectional studies (Figure 2A), all demonstrated strengths in defining their research questions and outcome measures; however, several lacked reporting on key elements such as participation rates and follow-up adequacy, indicating potential risk of bias. The single case–control study [32] (Figure 2B) was generally well conducted but showed weaknesses in reporting on population clarity and confounding controls. The five controlled intervention studies (Figure 2C) varied in quality, with some showing solid randomization and outcome measurement practices, while others lacked detailed reporting on method of randomization, blinding, allocation concealment, and participant retention. Overall, these findings suggest a moderate risk of bias across most included studies, primarily due to insufficient reporting and methodological limitations.

### 3.4. Topic Identification

The LDA algorithm enabled us to identify two topics related to VD during the transition from acute to chronic pain, which are presented in this section. We develop the discussion starting from Topic 1, as it deals with the impact of VD levels, and then treat Topic 2, which is more specific, as it examines the influence of VD supplementation.

#### 3.4.1. Topic 1: VD Levels and Its Relationship with Transition from Acute to Chronic Pain

This topic includes eight studies, the majority of which are observational in nature, including cross-sectional, prospective, retrospective, cohort, and case–control designs [30,31,32,33,37,38,39,42]. These studies examined the association between circulating levels of vitamin D (VD) and pain conditions, specifically focusing on whether VD deficiency or insufficiency may influence the transition from acute to chronic pain. Conditions explored include postoperative pain, chemotherapy-induced peripheral neuropathy (CIPN), chronic lower back pain, and chronic widespread pain.

By examining the top 30 most significant terms and their frequency within the eight papers categorized under this topic, it became apparent that the fundamental aspect of Topic 1 was the relationship between VD blood/serum levels and acute, subacute, or chronic pain conditions. These studies aim to understand whether higher or insufficient levels of VD could potentially affect the process of chronitization of pain in conditions frequently associated with the transition from acute to chronic pain. Studies clustered in Topic 1 and their main results are summarized in Table 3.

#### 3.4.2. Topic 2: The Impact of VD Supplementation on the Transition from Acute to Chronic Pain

This topic includes six studies that investigated the effects of vitamin D (VD) supplementation on the progression from acute to chronic pain and associated outcomes. Five of the studies were randomized controlled trials (RCTs) [34,35,36,40,41], while one, by Prieto-Alhambra et al. [43], was an observational prospective cohort study.

VD supplementation was observed to have a positive effect on quality of life and a reduction in pain [45]. Studies clustered in Topic 2 and their main results are summarized in Table 4. These studies examined various clinical settings, such as post-operative pain, aromatase inhibitor-induced arthralgia, and musculoskeletal pain. All included studies monitored serum VD levels throughout the intervention period.

## 4. Discussion

The mechanisms underlying pain chronification are multifactorial and complex, encompassing inflammatory and neuropathic processes as well as genetics and mental status [46,47]. Theoretically, preventing or reversing the pathological changes during the transition from acute to chronic pain has the potential to prevent or minimize the development of chronic pain. Although any painful condition can lead to the chronification of pain, it is particularly common with surgical trauma [48], lower back pain [17,49,50], and osteoarthritis [51]. Several attempts have been made to develop an analgesic approach to prevent this transition, but available evidence is limited so far [52]. Monotherapy often leads to insufficient therapeutic response; in addition, treating acute pain aggressively to avoid persistent pain is not without risks, such as the occurrence of side effects, the unneeded use of opioids, and the risk of chronic opioid use [7]. Compelling evidence has shown the potential of VD in exerting an influence on pain manifestation, thereby playing a role in the etiology and maintenance of chronic pain states and associated comorbidities [53]. VD is also thought to be of clinical benefit in treating chronic pain without the side effects of currently available analgesics, although its efficacy in specific pain conditions needs further investigation [52,53,54]. The purpose of this systematic review was to summarize the available clinical evidence on the impact of VD in the pain transition process and to evaluate the use of VD supplementation as a potential strategy in preventing or limiting the pain chronification process. In this frame, Zeng et al. demonstrated that low VD levels among patients undergoing video-assisted thoracoscopic surgery are associated with increased moderate to severe postoperative pain within 48 h compared to those with sufficient VD levels [30], while no significant differences in static or dynamic pain scores were detected at 3 months after surgery. In addition, a study conducted by Lee et al. found that nearly half of the patients undergoing knee arthroplasty had low preoperative VD levels, which were associated with increased pain intensity [33]. Lower VD levels did not influence morphine consumption or the quality of postoperative recovery compared to patients with sufficient VD levels. However, patients with VD deficiency may be more prone to experiencing more severe acute and chronic persistent pain after surgery [33], suggesting a possible role of this hormone as a predictive marker of pain intensity. In this context, promising results have been reported by Melika et al., showing that patients exposed to VD for a longer time before the operative time had an insignificantly lower pain score after craniotomy [34]. In addition, Krasowska and colleagues report that supplementation with VD enhanced the reduction in systemic inflammation markers, and when combined with surgery and early postsurgical rehabilitation it may decrease the intensity of pain in LBP patients undergoing PLIF [35]. Results on the increase in pain intensity linked to VD deficiency have also been reported in patients with acute and chronic non-specific LBP by Hao-Wei et al. However, the authors did not rule out a possible role of VD in the transition from acute LBP to chronic LBP owing to the hormone’s well-established effect on neuroplasticity [42]. Although VD insufficiency did not directly influence the transition from acute to chronic pain, the evidence that it affects pain intensity is clinically relevant. In chirurgical settings, pain intensity represents a risk factor for chronicity [48], and the pharmacological approach to controlling acute postoperative pain involves aggressive therapy encompassing the administration of a systemic opioids, non-steroidal anti-inflammatory drugs, and the delivery of local anesthetic to peripheral afferents supplying the chirurgical field. Acute to chronic pain transition may also occur as an adverse effect of chemotherapy [55]. Up to 70% of patients who underwent therapy with paclitaxel developed so-called chemotherapy-induced peripheral neuropathy (CIPN), which profoundly affects the quality of life [52,55]. Of note, around 30% of patients will still have CIPN a year or more after finishing chemotherapy [38]. In this population, VD deficiency did not affect the pain chronification process but rather represents a potential risk factor for both developing CIPN and severe pain [32,38,39]. By contrast, conflicting results have been reported in joint arthralgia related to aromatase inhibitor therapy. Indeed, Niravath et al. showed no significant beneficial effects of high-dose VD supplementation for pain development prevention in this clinical setting [40]. By contrast, Khan et al. found that higher VD levels (> 66 ng/mL) correlated with less joint pain and disability in patients who underwent aromatase inhibitor therapy [41]. Similarly, other studies have also shown some benefits of high-dose VD replacement [35]. Rastelli et al. reported that pain decreased significantly at 2 months in the VD group compared to placebo. The effect on pain was not observed at 4 and 6 months when the majority of subjects were switched from weekly to monthly VD supplementation [36]. In addition, Prieto-Alhambra et al. concluded that a target concentration of 40 ng/mL VD may prevent the development of aromatase-induced arthralgia, but higher loading doses are required to attain this level in women with a deficiency at baseline [43]. It is worth noting that this threshold exceeds the target of 20 ng/mL recommended by the 2010 Institute of Medicine (IOM) report [54]. Although the results of the current systematic review have been mixed, the studies underscore the multifaceted role of VD in various pain conditions, including acute and subacute lower back pain (LBP), chronic widespread pain (CWP), chemotherapy-induced peripheral neuropathy (CIPN), and aromatase inhibitor-induced arthralgia. However, it is important to note that large-scale randomized controlled trials have not consistently supported a benefit of VD supplementation on pain outcomes. For example, the VITAL-Pain ancillary study to the VITAL trial evaluated the effect of long-term supplementation with vitamin D (2000 IU/day) and omega-3 fatty acids (1 g/day) in over 19,000 older adults. The study found no significant reduction in pain prevalence or severity after a median follow-up of 5.3 years, compared to placebo [56]. These findings suggest that while observational and small-scale studies may indicate a potential role of VD in pain modulation, supporting the view that the role of high levels of vitamin D prevents chronic disease [57], the clinical impact of supplementation, particularly at moderate doses, remains uncertain in the general aging population. This further emphasizes the need to tailor interventions based on specific patient characteristics, baseline VD levels, and the etiology of pain. Nevertheless, maintaining adequate VD levels may still offer benefits in managing pain or reducing its intensity across various clinical contexts, which are themselves risk factors for the transition from acute to chronic pain. Further research is warranted to clarify VD’s role in pain management and to determine whether targeted supplementation can meaningfully influence the incidence or severity of painful conditions.

### Limitation of the Study

This study has several limitations that must be acknowledged. The majority of the included studies are observational in nature and therefore susceptible to inherent biases. First, the inability to control for all potential confounding factors, such as physical activity, comorbidities, diet, and socioeconomic status, may have introduced bias. Second, reverse causation cannot be ruled out, particularly in cross-sectional studies, where pain conditions might lead to reduced sun exposure and subsequent vitamin D deficiency. Third, differences in serum vitamin D assay methods and pain assessment tools may affect the reliability and comparability of findings across studies. Additionally, significant heterogeneity exists among the included studies in terms of study design, outcome measures, and the populations examined. This variability further limits the ability to synthesize findings and draw consistent conclusions. Although observational data suggest a potential association between vitamin D status and pain outcomes, a direct causal relationship has not been established. The current findings are hypothesis-generating and highlight the need for well-designed, high-quality randomized controlled trials (RCTs) to determine whether vitamin D plays a role in preventing or mitigating the transition from acute to chronic pain. Moreover, a major limitation of this review is the limited number of studies that specifically investigate vitamin D in the context of the transition from acute to chronic pain. This scarcity of data precluded the performance of a meta-analysis.

## 5. Conclusions

The transition from acute to chronic pain is a complex and multifactorial event involving various pathophysiological changes and is influenced by factors such as individual susceptibility, the duration and intensity of the initial pain, and the effectiveness of therapeutic approaches. In this scenario, VD emerges as a potential pain modulator, with evidence suggesting a role in reducing the intensity of acute pain and preventing some forms of persistent pain. Our qualitative analysis indicated that VD deficiency is related to increased postoperative pain intensity and a greater propensity to develop chronicity in conditions such as lower back pain, arthrosis, and chemotherapy neuropathy. However, its direct involvement in the transition from acute to chronic pain remains controversial. Despite the discrepancies, it is essential to assess the status of VD in patients at risk and consider its integration as part of a multimodal therapeutic strategy, especially in surgical settings. In this context, a preexisting VD deficiency is associated with a higher risk of persistent post-surgical pain, thus favoring its chronification. Further randomized clinical trials (i.e., double-blind and placebo-controlled, considered the “gold standard” of clinical studies) to assess VD and its influence on the transition from acute to chronic pain are warranted.

## Figures and Tables

**Table 3:** List of papers clustered in Topic 1.

Study/Year	Results
Chen et al., 2023 [38]	Of 1191 women, those with pre-treatment VD insufficiency (33.3%) had a higher risk of severe CIPN (20.7% vs. 14.2%; OR 1.57; 95% CI: 1.14–2.15; *p* = 0.005).
Zeng et al., 2022 [30]	Multivariable analysis showed that patients with low VD levels had a significantly higher risk of acute moderate–severe post-operative pain (OR 2.44; 95% CI: 1.18–5.04; *p* = 0.016), though pain scores at 3 months did not differ by vitamin D status.
Hao-Wei et al., 2021 [42]	Spearman’s analysis showed a significant negative correlation between vitamin D and IL-6 levels in both Ns-ALBP (r = −0.158, *p* = 0.027) and Ns-CLBP groups (r = −0.426, *p* < 0.001). VD levels also negatively correlated with VAS scores in Ns-CLBP patients (r ≈ −0.31, *p* < 0.001), but not in those with Ns-ALBP (*p* > 0.05).
Jennaro et al., 2020 [39]	Patients with VD deficiency showed a greater increase in neuropathic pain (36 ± 23 vs. 16 ± 16; *p* = 0.003) and a non-significant trend toward higher risk of treatment disruption (OR 2.98; 95% CI: 0.72–12.34; *p* = 0.16). Multivariable analysis confirmed an inverse association between baseline vitamin D levels and pain severity (β = −0.04; *p* = 0.02).
Panwar et al., 2018 [31]	While overall vitamin D deficiency rates were similar across CLBP, SLBP, and controls (~51%), both CLBP and SLBP groups had a significantly higher proportion of individuals with moderate to severe deficiency (≤16 ng/mL) compared to controls (CLBP: 43.6%, SLBP: 43.5%, controls: 20.1%; *p* < 0.001 and *p* = 0.001, respectively). This suggests a potential link between more severe vitamin D deficiency and chronic or subacute low back pain.
Grim et al., 2017 [32]	The key finding was that pre-chemotherapy vitamin D supplementation may offer neuroprotection against CIPN, as patients who developed CIPN consistently had lower 25(OH)D levels during the study period.
McCabe et al., 2016 [37]	At follow-up, 6.5% of participants developed new chronic widespread pain (CWP), while 24.9% remained pain-free. After adjusting for confounders, individuals in the lowest quintile of 25(OH)D (<15.6 ng/mL) had a nearly twofold increased risk of developing CWP compared to those in the highest quintile (≥36.3 ng/mL) (OR = 1.93; 95% CI: 1.0–3.6).
Lee et al., 2015 [33]	Patients with preoperative vitamin D deficiency were more likely to experience moderate-to-severe persistent postoperative pain (13.8% vs. 5.9%; *p* = 0.05). This deficiency was significantly associated with increased risk of persistent pain (OR 2.64; 95% CI: 1.03–6.77; *p* = 0.04).

Abbreviations: 25(OH)D, 25-hydroxyvitamin D; CI, confidence interval; CIPN, chemotherapy-induced peripheral neuropathy; CLBP, chronic lower back pain; CWP, chronic widespread pain; IL-6, Interleukin-6; Ns-ALBP, non-specific acute lower back pain; Ns-CLBP, non-specific chronic lower back pain; OR, odds ratio; SLBP, subacute lower back pain; VAS, Visual Analog Scale; VD, vitamin D.

**Table 4:** List of papers clustered in Topic 2.

Study/Year	Results
Melika et al., 2019 [34]	Did not find any significant effect of VD. However, a longer time before the operative time had an insignificantly lower pain score.
Krasowska et al., 2019 [35]	No significant difference in pain intensity was observed between VD and placebo groups after 5 weeks of supplementation. However, both groups showed a marked reduction in VAS scores following surgery and rehabilitation (*p* < 0.006 to *p* < 0.0001). Pain improvement was more pronounced in the VD group compared to placebo, both post-surgery (VAS: 2.83 ± 0.51 vs. 3.29 ± 0.37) and after rehabilitation (1.28 ± 0.29 vs. 2.62 ± 0.47).
Niravath et al. (2019) [40]	High-dose VD effectively increased serum levels, but it did not significantly impact the incidence of aromatase inhibitor-induced arthralgia. Additionally, neither baseline nor 12-week VD levels predicted the development of this condition.
Prieto-Alhambra et al. (2011) [43]	Following supplementation, half of the patients did not reach adequate 25(OH)D levels by 3 months. Overall, joint pain increased (mean change: +1.16 ± 2.66; *p* < 0.001), but this increase was significantly less in those who achieved ≥40 ng/mL of 25(OH)D (*p* = 0.02), with a lower risk of developing arthralgia (OR 0.12; 95% CI: 0.03–0.40). Achieving this target level may help prevent aromatase inhibitor-induced arthralgia, though higher loading doses are needed for those initially deficient.
Rastelli et al. (2011) [36]	At 2 months, patients receiving high-dose VD (HDD) showed significantly greater improvements in pain scores compared to placebo, including FIQ pain (*p* = 0.0045), BPI worst pain (*p* = 0.04), average pain (*p* = 0.0067), pain severity (*p* = 0.04), and pain interference (*p* = 0.034). The beneficial effects of HDD on aromatase inhibitor-associated musculoskeletal symptoms (AIMSSs) were more pronounced in Stratum B than Stratum A across all time points.
Khan et al. (2010) [41]	At baseline, the median 25(OH)D level was 27 ng/mL (range: 9–61 ng/mL). VD deficiency (≤20 ng/mL) was present in 30% of participants, while an additional 33% had insufficient levels (21–31 ng/mL).

Abbreviations: 25(OH)D, 25-hydroxyvitamin D; AIMSS, Aromatase Inhibitor-Associated Musculoskeletal Symptom; BPI, Brief Pain Inventory; CI, confidence interval; FIQ, Fibromyalgia Impact Questionnaire; HDD, high-dose vitamin D; OR, odds ratio; VAS, Visual Analog Scale; VD, vitamin D.

## Data Availability

All data generated/analyzed throughout this research are included in this article.

## Abbreviation

VD	Vitamin D
MySLR	My Systematic Literature Review
LDA	Latent Dirichlet Allocation (algorithm for topic modeling)
NRS	Numeric Rating Scale
VAS	Visual Analog Scale
BPI	Brief Pain Inventory
BPI-SF	Brief Pain Inventory–Short Form
MODQ	Modified Oswestry Disability Questionnaire
MNSI	Michigan Neuropathy Screening Instrument
WOMAC	Western Ontario and McMaster Universities Osteoarthritis Index
CIPN	Chemotherapy-induced peripheral neuropathy
CIPN20	Chemotherapy-Induced Peripheral Neuropathy 20-item Quality of Life Questionnaire
FIQ	Fibromyalgia Impact Questionnaire
HAQ	Health Assessment Questionnaire
HAQ-DI	Health Assessment Questionnaire–Disability Index
AI	Aromatase inhibitor
AIMSS	Aromatase Inhibitor-Associated Musculoskeletal Symptom
PL	Placebo
IU	International Units
25(OH)D	25-hydroxyvitamin D (a marker for Vitamin D status)
NP	Neuropathic pain
CWP	Chronic widespread pain
NAAA	N-Acylethanolamine acid amidase
SRE	Summary Risk of Bias Evaluation
